# ChAd155-RSV vaccine is immunogenic and efficacious against bovine RSV infection-induced disease in young calves

**DOI:** 10.1038/s41467-022-33649-3

**Published:** 2022-10-17

**Authors:** Rineke de Jong, Norbert Stockhofe-Zurwieden, Judith Bonsing, Kai-Fen Wang, Sarah Vandepaer, Badiaa Bouzya, Jean-François Toussaint, Ilse Dieussaert, Haifeng Song, Ann-Muriel Steff

**Affiliations:** 1 Wageningen Bioveterinary Research, Wageningen University & Research, Houtribweg 39, 8221 RA Lelystad, The Netherlands; 2 GSK, 14200 Shady Grove Road, Rockville, MD 20850 USA; 3 CONSULTYS Benelux S.A, 73D Rue de Namur, 1000 Brussels, Belgium; 4 GSK, Rue de l’Institut 89, 1330 Rixensart, Belgium; 5 Present Address: Atara Biotherapeutics, Inc., 2380 Conejo Spectrum St Suite 200, Thousand Oaks, CA 91320 USA; 6 Present Address: Sanofi-Pasteur, 14 Espace Henry Vallée, 69007 Lyon, France; 7 Present Address: Suzhou Abogen Bioscience Ltd, Suzhou, Jiangsu China

**Keywords:** DNA vaccines, Viral infection, Antibodies

## Abstract

Respiratory syncytial virus (RSV) infection causes a substantial lower-respiratory-tract disease burden in infants, constituting a global priority for vaccine development. We evaluated immunogenicity, safety and efficacy of a chimpanzee adenovirus (ChAd)-based vaccine candidate, ChAd155-RSV, in a bovine RSV (bRSV) challenge model. This model closely reproduces the pathogenesis/clinical manifestations of severe pediatric RSV disease. In seronegative calves, ChAd155-RSV elicits robust neutralizing antibody responses against human RSV. Two doses protect calves from clinical symptoms/lung pathological changes, and reduce nasal/lung virus loads after both a short (4-week) and a long (16-week) interval between last immunization and subsequent bRSV challenge. The one-dose regimen confers near-complete or significant protection after short-term or long-term intervals before challenge, respectively. The presence of pre-existing bRSV-antibodies does not affect short-term efficacy of the two-dose regimen. Immunized calves present no clinical signs of enhanced respiratory disease. Collectively, this supports the development of ChAd155-RSV as an RSV vaccine candidate for infants.

## Abstract (web summary)

A pediatric RSV vaccine is an unmet medical need, even after >50 years of effort. Here, the authors show that a chimpanzee adenovirus based RSV vaccine candidate protects calves from disease upon RSV infection, regardless of the time after vaccination (1 or 4 months) or the presence of maternal antibodies.

## Introduction

Respiratory syncytial virus (RSV) infection is a major cause of lower respiratory tract disease (LRTD) in infants. Infants aged under 1 year have the highest incidence of severe LRTD (i.e., bronchiolitis and pneumonia)^1^, and the disease burden extends even beyond the second year of life. Worldwide, there were an estimated 33.1 million new episodes of RSV-linked acute lower respiratory infection in children aged under 5 years (in 2015), and about 10% of these cases were severe enough to warrant hospitalization^2^. In the same age group, the estimated RSV-attributable global mortality was around 59,600 in-hospital deaths and 118,200 overall deaths per year, 99% of which occurred in developing countries^2,3^.

Due to this high incidence of severe disease and a lack of cost-effective preventative measures, RSV represents a major priority for vaccine development^4^. However, progress in this area has been largely hindered by the possibility of vaccination-induced enhanced respiratory disease (ERD), which manifested after vaccination with a formalin-inactivated RSV vaccine candidate (FI-RSV, Lot 100) in the 1960s^5–7^. In one of these trials, FI-RSV vaccination of 31 infants resulted in 2 deaths and 80% hospitalization (of the 20 vaccinees who became infected), as compared to 5% hospitalization in the control group vaccinated with parainfluenza vaccines^5^. Although the mechanisms underlying FI-RSV-induced ERD are not completely clear, many studies agreed that a T helper (Th) 2-biased cellular response, a high ratio of non-functional antibodies (Abs) to neutralizing Abs (nAbs), and a lack of CD8^+^ cytotoxic T cell responses are among its major attributes^8–10^. An ideal RSV candidate should therefore induce high nAb titers, a Th1-biased CD4^+^ T cell response, and CD8^+^ T cell responses. Consequently, RSV vaccines that induce cellular expression of RSV antigens, such as viral vectored or nucleic acid-based platforms, are considered particularly suited for RSV pediatric vaccination^11^. These candidates may behave biologically as a live-virus vaccination as the antigens are produced intracellularly, thus preferentially inducing CD8^+^ T cell responses and skewing the adaptive immunity towards a Th1 phenotype.

Adenoviral (Ad) vectors represent an attractive vaccine platform, as they are known to induce both cellular and humoral responses against the expressed antigens^12–14^. Particularly adenoviruses of simian origin, such as chimpanzee Ad (ChAd) strains, are commonly used as vaccine vectors, due to their lower seroprevalence and thus decreased vector neutralization in humans as compared to human Ad5^15–20^. Several ChAd-vectored candidate vaccines against infectious agents including RSV and SARS-CoV-2 have been demonstrated to induce favorable immunogenicity, safety and efficacy profiles in clinical trials^21–25^. A pediatric RSV vaccine candidate based on a group C ChAd 155 vector, ChAd155-RSV, encodes RSV fusion protein (F) in secreted form to induce humoral responses, and intracellularly expressed transcription anti-termination protein M2-1 and nucleocapsid protein N aimed to induce a CD8^+^ T cell response^22,26^. ChAd155-RSV has first been evaluated in animal models^26^ and has subsequently progressed to Phase I, I/II, and II clinical development stages^22^ (NCT02927873, NCT03636906).

Preclinical studies in RSV infection models indicated that no single validated animal model can faithfully predict the clinical outcome and efficacy of an RSV vaccine candidate in the human target population^27–29^. Bovine RSV (bRSV) infection symptoms in young calves share many aspects of severe RSV infection in human infants with respect to pathogenesis and clinical manifestations, including fever, runny nose, coughing and labored breathing^27,30,31^. The experimental calf model is thus a unique translational model in the preclinical evaluation of human RSV vaccines with regard to clinical efficacy^32^, conferring advantages over mice and cotton rats model which are only semi-permissive for the human challenge virus, requiring a high challenge dose (i.e., 5-6 log_10_ PFU/TCID_50_)_,_ and do not show obvious respiratory symptoms^27,33–36^. Human and bovine RSV proteins including the F protein have a high-level homology with >80% amino acid identity, thus inducing cross-reactive immunity^27^. In addition, FI-bRSV-induced ERD in calves has been reported by several groups^30,37–39^, allowing the identification of ERD signs already in the preclinical vaccine development phases. In the current study, the humoral immunogenicity, safety, and efficacy of the ChAd155-RSV vaccine has been evaluated in a bovine RSV challenge model in young calves. We report that ChAd155-RSV vaccination elicited nAb responses, and two doses protected the calves from clinical symptoms and lung pathological changes upon bRSV challenge. The presence of pre-existing RSV-antibodies did not affect the short-term efficacy of a two-dose vaccination regimen in this model, and no clinical signs of ERD were observed post-challenge. The collective data supported further development of the ChAd155-based RSV vaccine as a candidate for pediatric immunization.

## Results

**Fig. 1:**
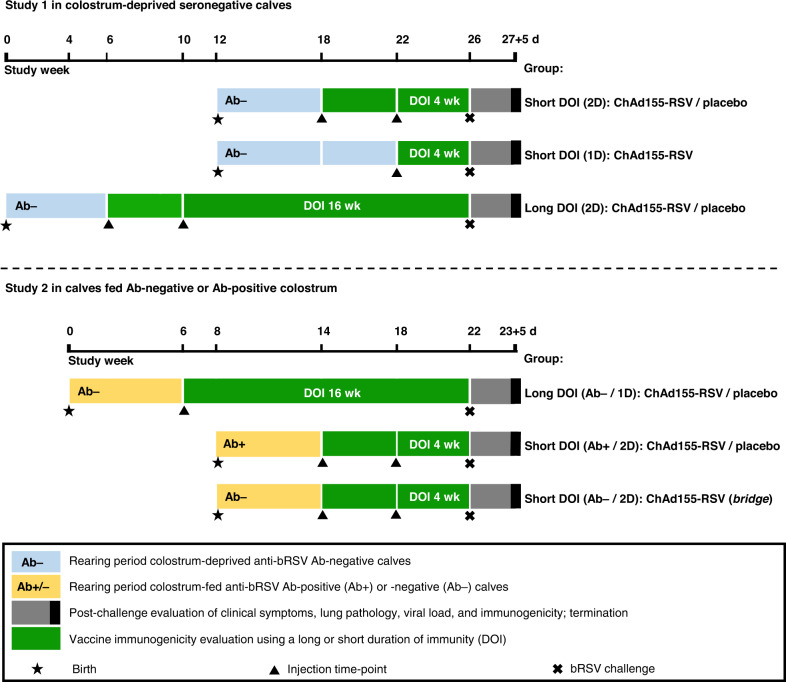
Study designs. Study 1 was conducted in bovine RSV (bRSV)-seronegative (Ab−) colostrum-deprived calves. Study 2 was conducted in Ab− calves fed bRSV antibody-negative colostrum, and in bRSV-seropositive (Ab+) calves fed Ab-positive colostrum. Animals (*n* = 7 to 9/group) received 1 or 2 doses (1D or 2D, respectively) of either ChAd155-RSV vaccine or placebo (phosphate-buffered saline; controls). Corresponding vaccine and control groups followed the same treatment/challenge schedules after either a short (4 weeks) or a long (16 weeks) duration of immunity (short DOI or long DOI, respectively). Short or long DOI calves were recruited in two separate cohorts to arrange the challenge on the same calendar day.

In two separate studies (Fig. 1 and Table 1), the protective efficacy of a single-dose or two-dose vaccination regimen with ChAd155-RSV was assessed upon bRSV challenge. The challenge was performed after either a short (4-week) or a long (16-week) interval after the (last) vaccination—further referred to as a short/long duration of immunity (DOI). In addition, the efficacy of a two-dose regimen was assessed in the presence of pre-existing bRSV-antibodies after a short DOI.

**Table 1:** Detailed study designs

Study	Colostrum feeding^a^	Sero-status^a^	Treatments			DOI (weeks) before challenge^d^	*N*
			Test article^b^ (i.m.; 2 mL/dose)	No. of doses	Age at vaccine administration (weeks)^c^		
1	No	–	ChAd155-RSV	2	6, 10	4	8
	No	–	Placebo	2	6, 10	4	8
	No	–	ChAd155-RSV	1	10	4	8
	No	–	ChAd155-RSV	2	6, 10	16	8
	No	–	Placebo	2	6, 10	16	7
2	Yes	Ab−	ChAd155-RSV	1	6	16	9
	Yes	Ab−	Placebo	1	6	16	9
	Yes	Ab+	ChAd155-RSV	2	6	4	9
	Yes	Ab+	Placebo	2	6	4	9
	Yes	Ab−	ChAd155-RSV	2	6	4	9

^a^In Study 1, calves received only antibody-negative milk replacer. In Study 2, a single feeding with anti-bRSV antibody-negative/-positive (Ab−/+) colostrum was supplied followed by feeding with antibody-negative milk replacer.

^b^ChAd155-RSV at 5 × 10^10^ viral particles/dose or phosphate-buffered saline, given by intramuscular (i.m.) injection.

^c^All animals in a given group were born within a 3–4-day period.

^d^Duration of immunity (DOI) period (weeks since last vaccine administration) was followed by bRSV challenge at 3.2 (Study 1) or 3.7 (Study 2) log_10_ TCID_50_/2 mL dose. Necropsy was performed on days 12/13 post challenge for all groups.

### ChAd155-RSV efficacy against bRSV-induced clinical disease

**Fig. 2:**
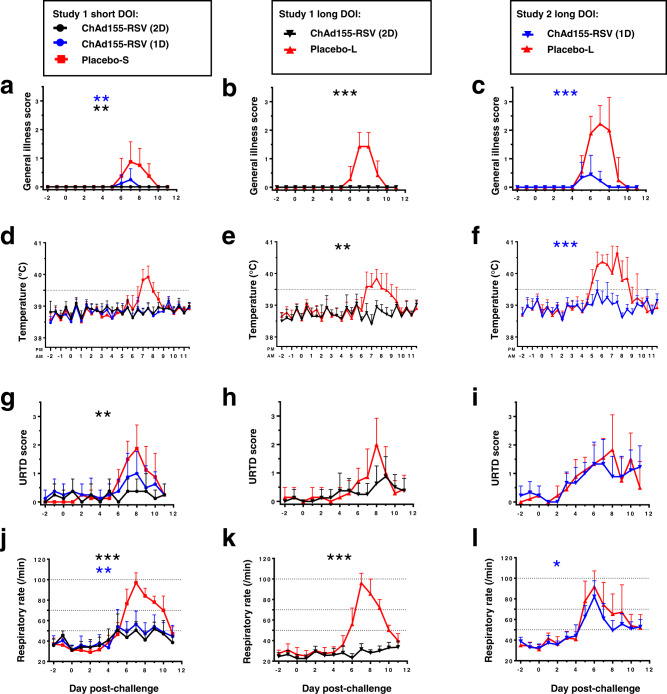
Clinical symptoms. Calves (*n* = 7–9 biologically independent animals, see Table 1) received either a single or two doses (1D or 2D, respectively) of ChAd155-RSV vaccine or placebo, followed by a short (4 weeks) or long (16 weeks) duration of immunity (short DOI or long DOI, respectively), and then a bovine RSV challenge. Placebo-S and placebo-L groups indicate the control groups subjected to the short-DOI and long-DOI regimens, respectively (note that the sample size [*n* = 9] of the placebo-L group of Study 2 decreased to *n* = 5 on day post-challenge [dpc] 8, and to *n* = 4 on dpc 9–11; see Fig. 3). Clinical symptoms in the ChAd155-RSV-immunized or placebo-treated calves were evaluated according to the scoring system presented in Supplemental Table 1 before and after the challenge at the time points indicated. Data are presented as means with 95% confidence intervals (vertical bars). Scores of general illness symptoms (**a**–**c**), rectal temperatures (**d**–**f**), upper respiratory tract disease (URTD) scores (**g**–**i**), and respiratory rates (**j**–**l**) are presented. Comparisons between the vaccine groups and the respective control groups were estimated using two-sided *t* tests on individual AUC data. *P ≤ 0.05; **P ≤ 0.01; ***P ≤ 0.001. Source data and statistical analyses with exact *P* values are provided as a Source data and Supplementary Data 1 and 2, respectively.

In Study 1, placebo control calves developed typical clinical symptoms of bRSV infection when challenged after a short DOI. General illness symptoms (expressed in scores) accompanied by fever (rectal temperature >39.5 °C) appeared at day 6 post challenge (dpc 6), peaked at dpc 7/8 and resolved by dpc 10/11 (Fig. 2a, d). Simultaneously, their upper respiratory tract disease (URTD) scores and respiratory rates were increased (Fig. 2g, j). In contrast, calves having received 2 doses of the vaccine were nearly completely protected from clinical symptoms, including fever. Although these animals occasionally showed (slightly) increased URTD scores and respiratory rates, these levels remained significantly lower than those for the controls (*P* ≤ 0.01 and *P* ≤ 0.001, respectively). As compared to the two-dose vaccine group, the single-dose vaccine group exhibited slightly higher general illness and URTD scores. However, the protective efficacy in this group was overall comparable to that in the two-dose group, and clearly higher relative to the control group.

Upon challenge after a long DOI (Fig. 2b, e, h, k), clinical symptoms in the controls were similar to those in the short-DOI controls. Two vaccinations completely prevented general illness, fever, and increased respiratory rates. However, mild URTD symptoms were observed on a limited number of days (Fig. 2h).

In Study 2, performed separately, the efficacy of a 1-dose vaccination for protection against bRSV challenge 16-weeks after immunization was evaluated (Fig. 2c, f, i, l). Unexpectedly, the challenge outcome in the age-matched placebo control group in this study was more severe than in Study 1. In Study 1, all placebo control calves developed moderately to severely increased breathing rates from dpc 6 onwards, and recovered by dpc 10 or 11. In Study 2, the onset of respiratory distress was observed earlier, at dpc 5, and most of the placebo control calves developed severely increased breathing rates (Fig. 2l). This resulted in persistent respiratory distress or non-reversible respiratory failure in five (out of nine) placebo control calves. On dpc 7 and 8, four calves from the placebo group were pre-terminated as they complied to humane endpoint criteria while another calf had died unexpectedly at dpc 8. Despite the more severe clinical outcome of bRSV challenge in Study 2, the 1-dose vaccination group was nearly completely protected after long-term DOI against general illness and fever (*P* ≤ 0.001), while the breathing rate was slightly reduced (*P* ≤ 0.05) and the URTD symptoms were comparable to those in the placebo group (Fig. 2c, f, i, l). Nevertheless, none of the 1-dose ChAd155-RSV vaccinated calves was pre-terminated, and all of them had nearly recovered by the end of the study (dpc 12).

### Effect of ChAd155-RSV on bRSV-induced lung pathology

**Fig. 3:**
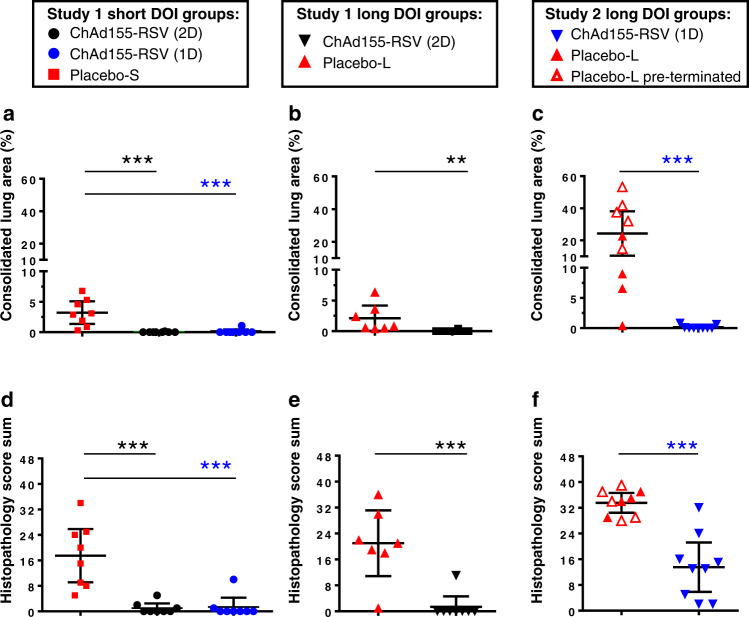
Lung pathology. Calves (*n* = 7 to 9 biologically independent animals, see Table 1) received a single or two doses (1D or 2D, respectively) of ChAd155-RSV vaccine or placebo, followed by a short (4 weeks) or long (16 weeks) duration of immunity (short DOI or long DOI, respectively) and then a bovine RSV challenge. Placebo-S and placebo-L groups indicate the control groups subjected to the short-DOI and long-DOI regimens, respectively. On day post-challenge (dpc) 12/13 animals were subjected to necropsy. Macroscopic analysis of the consolidated lung areas (CLAs; **a**–**c**) and sums of lung pathology scores determined by microscopic analysis of the lung pathological changes (**d–f**) are presented for the study groups indicated in the keys above the graphs. Sums of lung pathological scores were derived by addition of the scores for alveolitis, interstitial pneumonia, peribronchitis and bronchitis per individual animal. Data are presented as means ± 95% confidence intervals. Each symbol represents an individual animal. Open symbols in **c**, **f** indicate animals that were pre-terminated or found dead on dpc 7/8. Vaccine groups were compared by two-sided *t* tests with the respective control groups using one-way ANOVA for CLAs and histopathology score sums with treatment as fixed effect and the appropriate variance assumption. Significant differences are indicated by asterisks (**P ≤ 0.01; ***P ≤ 0.001). Source data and statistical analyses with exact *P* values are provided as a Source data and Supplementary Data 1 and 2, respectively.

At necropsy (dpc 12/13), lungs were examined macroscopically to determine the consolidated lung area (CLA) as percentage of the total lung area (see Supplemental Fig. 1 for representative photos, and Fig. 3a–c). Additionally, three tissue samples per lung were examined microscopically for histopathological changes (see Supplemental Fig. 2 for representative images), and sections were scored for evidence of bronchitis, peribronchitis/perivasculitis, interstitial pneumonia and alveolitis. Scores are presented as total histopathological sum scores (Fig. 3d–f) as well as by individual pathological symptom (Supplemental Fig. 3).

In Study 1, macroscopic CLAs upon short DOI and challenge were observed in all control calves (≤7%), but were only minimally or not observed in vaccinated calves of the single- or two-dose groups (Fig. 3a). All control calves displayed histopathological changes (scores 5 to 34, on a scale of 48; Fig. 3d). These changes varied from focal bronchitis/alveolitis to multifocal or extended changes associated with obliterating bronchitis and severe pneumonia, with an influx of neutrophil granulocytes and lymphomonocytic inflammatory cells in the alveoli. By contrast, vaccinated calves displayed minimal alterations (scores ≤5), except for one animal from the single-dose group with a score of 10 (Fig. 3d). In parallel, all seven control calves with long DOI showed CLAs of up to 6.37 %, whereas consolidation was absent (*n* = 7) or minimal (*n* = 1) in calves which received two vaccine doses (Fig. 3b). All control calves showed histopathological changes (scores: 1–36), while all except one (with score 11) in the two-dose vaccine group were completely protected against histopathological alterations (Fig. 3e).

In line with the clinical observations, lung pathology was more severe after the long DOI in Study 2. Indeed, compared to Study 1, control calves had larger CLAs and more histopathological changes (0.4–53% and scores 28–39; Fig. 3c, f, respectively). In addition, greater CLAs were observed in the five animals that were either pre-terminated (*n* = 4) or found dead (*n* = 1) at dpc 7/8 (Fig. 3c). The single-dose vaccine group exhibited at most minimal (0.8%) macroscopic consolidation, though microscopic evaluation still revealed histopathological changes (scores 2–32).

### ChAd155-RSV efficacy on bRSV replication in nasal and lung fluids

**Fig. 4:**
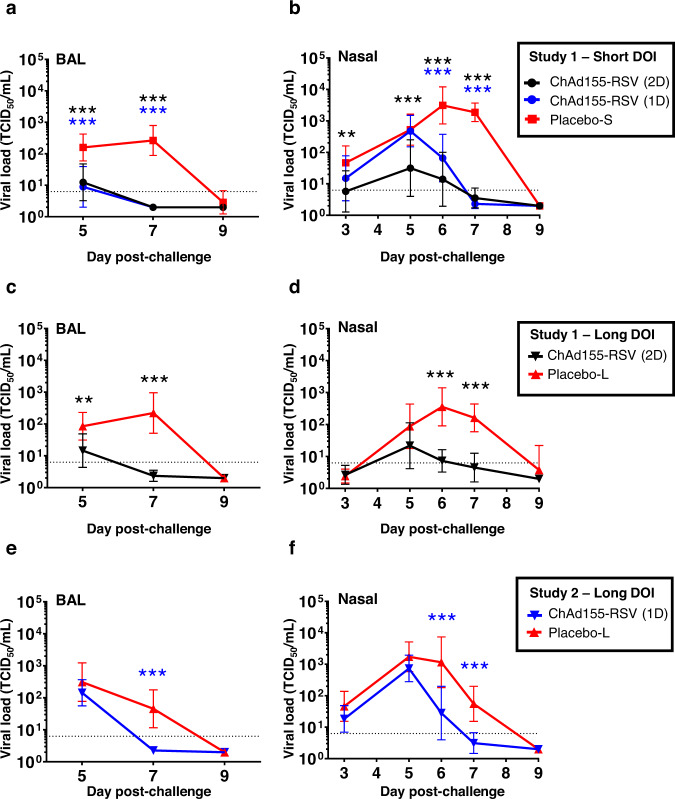
Viral loads. Calves (*n* = 7 to 9 biologically independent animals, see Table 1) received either a single or two doses (1D or 2D, respectively) of ChAd155-RSV vaccine or placebo, followed by a short (4 weeks) or long (16 weeks) duration of immunity (short DOI or long DOI, respectively), and then a bovine RSV challenge. Placebo-S and placebo-L groups indicate the control groups subjected to the short-DOI and long-DOI regimens, respectively (note that the sample size in the placebo-L group of Study 2 had decreased to *n* = 4 on day post-challenge 9; see Fig. 3). Virus loads of lung broncho-alveolar lavage (BAL) samples (**a**, **c**, **e**) and nasopharyngeal samples (**b**, **d**, **f**) are presented as geometric mean concentrations with 95% confidence intervals. Dotted lines (6.3 TCID_50_/mL) represent the lower limit of detection (LOD). Negative samples were assigned a value of 1.995. Vaccine groups were compared by two-sided *t* tests with the respective control groups using ANOVA mixed models for repeated measurements. Significant differences are indicated by asterisks color-matched with the vaccine group indicated in the keys above the figure (**P ≤ 0.01; ***P ≤ 0.001). Source data and statistical analyses with exact *P* values are provided as a Source data and Supplementary Data 1 and 2, respectively.

Following bRSV challenge, nasopharyngeal brush samples were obtained daily, and BAL samples on dpc 5, 7, and 9 (Fig. 4). The viral loads determined by bRSV qPCR on all samples (Supplemental Fig. 4) were consistent with the viral infectivity data generated from selected time points (described below).

In Study 1, virus loads in BALs from control calves of both the short- and long-DOI groups peaked on dpc 7 (mean titer ~300 TCID_50_/mL), but were cleared by dpc 9 (Fig. 4a, c). One or two vaccinations resulted in significantly lower viral loads at dpc 5 and 7 in both short-DOI groups (*P* ≤ 0.001) and in the two-dose long-DOI group (*P* ≤ 0.01 at dpc 5 and *P* ≤ 0.001 at dpc 7). Kinetics in the nasopharynx of short- or long-DOI controls were similar as for BALs, with a gradual increase from dpc 3 onwards, peaks on dpc 6 or 7 (mean titers:1000–10,000 TCID_50_/mL), and no re-isolated infectious virus on dpc 9 (Fig. 4b, d). Two vaccine doses significantly reduced viral loads as compared to controls, in both the short- and the long-DOI groups (on dpc 3, 5, 6 and 7, and on dpc 6 and 7, respectively). In the single-dose short-DOI vaccine group, viral clearance was also observed earlier than in controls, i.e., on dpc 6 and 7 (*P* ≤ 0.001, Fig. 4b).

In Study 2, the kinetics of viral replication were slightly advanced, with a peak at day 5 and decreased thereafter (Fig. 4e, f). This was likely due to the more virulent challenge. Nonetheless, a single vaccination resulted in earlier viral clearance compared to placebo, as observed in the lungs at dpc 7, and in the nasopharyngeal tract at dpc 6 or 7 (all *P* ≤ 0.001).

### ChAd155-RSV efficacy in the presence of pre-existing bRSV Abs

**Fig. 5:**
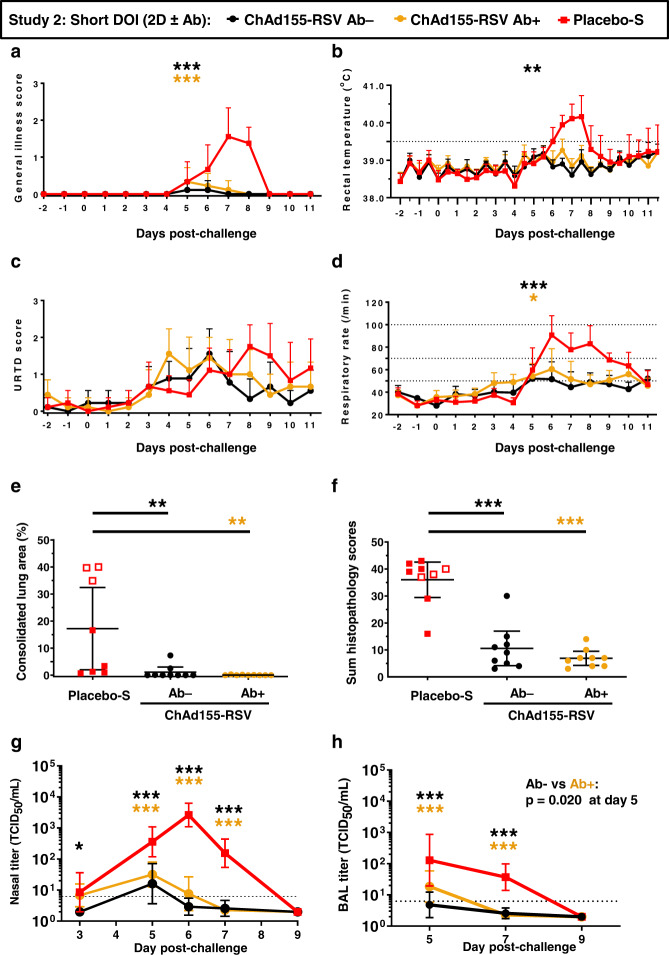
Impact of pre-existing bRSV Ab on ChAd155-RSV vaccine efficacy. In Study 2, calves (*n* = 9 biologically independent animals, see Table 1) with (Ab+) or without (Ab−) pre-existing antibodies derived from bovine RSV Ab+ or Ab− colostrum, received two doses (2D) of ChAd155-RSV or placebo, and were challenged after a short (4 weeks) duration of immunity (DOI). On day post-challenge (dpc) 12/13, animals were subjected to necropsy. The placebo-S group indicates the control group, which included 9 calves through dpc 7, decreasing to 8 calves on dpc 8, and then to 6 calves on dpc 9 to 11 (as the other calves were pre-terminated due to reaching humane endpoints). Analyzed were general illness scores (**a**), rectal temperatures (**b**), upper respiratory tract disease (URTD) scores, (**c**), respiratory rates (**d**), consolidated lung areas (CLAs; **e**), lung pathological changes at termination time point (**f**), and nasopharyngeal and lung viral loads (**g**, **h**, respectively; see Supplemental Table S1 for explanation on clinical scores). Differences between vaccine groups and the respective control groups were determined by two-sided ANOVA (with treatment as fixed effect and the appropriate variance assumption) performed either on the calculated areas under the curve (**a**–**d**) or on the single-timepoint data (**e**, **f**), or by ANOVA mixed models for repeated measurements (**g**, **h**). Means (**a**–**f**) or geometric means (**g**, **h**) with 95% confidence intervals (error bars; **a**–**h**) for each group are color-coded according to the keys in each graph. Open symbols in **e**, **f** indicate animals that were pre-terminated on dpc 7/8. Statistically significant differences are presented with asterisks color-matched with the vaccine group indicated in the key above the figure (*P ≤ 0.05; **P ≤ 0.01; ***P ≤ 0.001). Source data and statistical analyses with exact *P* values are provided as a Source data and Supplementary Data 1 and 2, respectively.

Most infants have maternal RSV Abs transferred via the placenta. We therefore investigated whether such pre-existing responses would impact the vaccine’s immunogenicity and efficacy. As transplacental transfer does not occur in the bovine species, newborn calves in Study 2 received colostrum, which was either bRSV Ab-positive (resulting in a group mean nAb titer of 1:200 at pre-vaccination), or bRSV Ab-negative. In both cases, colostrum administration was followed by two vaccine doses, 4 weeks apart, and a challenge after a short DOI (see Fig. 1 and Table 1). A control group with colostrum-derived pre-existing bRSV Ab received two phosphate-buffered saline (PBS) injections and a challenge following the same schedule. Upon challenge, this control group displayed the typical bRSV-induced clinical symptoms, including general illness, fever, increased respiratory rates and signs of URTD (Fig. 5a–d). Additionally, increases in CLAs, overall lung pathological scores and increased viral loads were observed in their BAL and/or nasal samples (Fig. 5e–h). Of note, this placebo group behaved similarly to the one-dose long-term DOI placebo group from Study 2, with more severe clinical symptoms compared to that in Study 1, leading to premature termination in 3 out of 9 animals. In contrast to the controls, calves in the two-dose vaccination groups with or without pre-existing bRSV Ab exhibited either a complete absence of symptoms, or significantly reduced symptoms of general illness, fever, and respiratory rates, although they still showed URTD symptoms (Fig. 5a–d). In addition, none of these vaccinated animals were pre-terminated. Although occasionally, clinical scores were slightly higher in the group with versus without pre-existing Abs, these differences were not statistically significant. Moreover, two vaccinations resulted in significant reductions in the CLAs—which were absent or minimal in all (9/9) animals with pre-existing Abs, and in nearly all (7/9) animals without pre-existing Abs—and in reduced pathology sum scores (*P* ≤ 0.01; Fig. 5e and *P* ≤ 0.001; Fig. 5f). The vaccinations also resulted in reduced virus titers and faster viral clearance from the lung and nasopharynx (Fig. 5g, h). Overall, the presence of pre-existing Abs had minimal impact on the vaccine efficacy in calves which received two vaccine doses and were challenged 4 weeks post-dose 2. Furthermore, the fact that all these animals (irrespective of the presence of pre-existing bRSV Ab) were protected from bRSV disease, as observed in Study 1 with the same two-dose short-DOI regimen (see Fig. 2), indicated that the vaccine performed similarly well after a more virulent challenge.

### ChAd155-RSV humoral immunogenicity

**Fig. 6:**
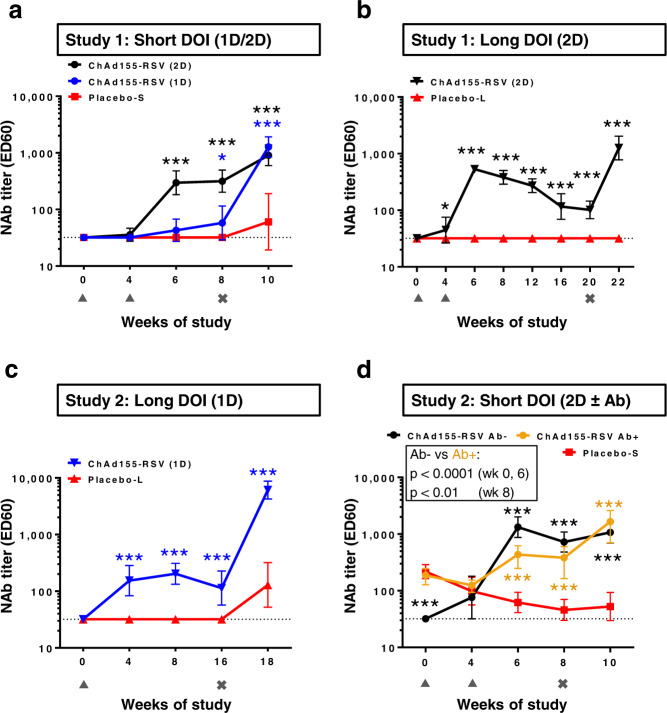
Human RSV A-specific nAb titers. Human RSV subtype A (hRSV A) neutralizing antibody (nAb) titers are presented. Titers are expressed as the inverse of the serum dilution causing 60% reduction in the number of plaques as compared to the virus control wells (ED_60_). Geometric means with 95% confidence intervals (error bars) for each group are color coded according to the keys in each graph. Calves (*n* = 7–9 biologically independent animals, see Table 1) received a single or two doses (1D or 2D, respectively) of ChAd155-RSV vaccine or placebo, followed by a short (4 weeks, **a**, **d**) or long (16 weeks, **b**, **c**) duration of immunity (short DOI or long DOI, respectively), and then a bovine RSV challenge. Placebo-S and placebo-L groups indicate the control groups subjected to the short-DOI and long-DOI regimens, respectively (note that the sample sizes of the placebo-L and placebo-S groups in Study 2 decreased post-challenge; see Figs. 3 and 5, respectively). Groups in **d** included calves with or without pre-existing bovine RSV Ab groups (Ab+ or Ab−, respectively). Weeks of study: weeks post-dose 1 for all groups except short DOI-1D, which was injected at week 4 (Study 1). Dotted lines represent the limit of detection (LOD) i.e., 32 ED_60_. Negative samples were assigned the value of the LOD. Titers of vaccine and control groups were compared by two-sided *t* tests using ANOVA mixed models for repeated measurements. Significant differences between vaccine groups and the respective control groups are presented as asterisks color-matched with the vaccine group indicated in the keys (*P ≤ 0.05; ***P ≤ 0.001). Triangles and crosses below the x-axis denote the time points of placebo or vaccine injections and the bRSV challenge, respectively. Individual values for all groups are presented in Supplemental Fig. 5. Source data and statistical analyses with exact P values are provided as a Source data and Supplementary Data 1 and 2, respectively.

Kinetics of vaccine-induced nAb responses were analyzed by standard neutralization assay using hRSV A Long. In Study 1 at 4 weeks post first vaccination, this vaccine dose had elicited low-level responses across the single-dose short-DOI group and both two-dose groups (geometric mean titers [GMTs]: 35.7–57.7; Fig. 6a, b and Supplemental Fig. 5a, b). Similarly, in Study 2, low titers were observed in the single-dose long-DOI group and in the two-dose group without pre-existing Abs (GMTs: 154 and 76, respectively; Fig. 6c, d and Supplemental Fig. 5c, d). However, the titer in the latter single-dose group was sustained at similar levels up to the time of challenge, i.e., 16 weeks after immunization.

In both studies, the second vaccination clearly increased the nAb titers (Fig. 6a, b, d, and Supplemental Fig. 5a, b, d). At 2 weeks post-dose 2, this was observed in the short- and long-DOI vaccine groups in Study 1 (GMTs: 296 and 531, respectively) and in the short-DOI vaccine group without pre-existing Abs in Study 2 (GMT: 1323). In Study 1, long-DOI, the titer gradually decreased over time, but remained detectable at 16 weeks post-dose 2 (GMT: 101; Fig. 6b and Supplemental Fig. 5b).

In Study 2, nAb responses induced by two vaccinations were also compared between calves with versus without pre-existing bRSV Abs (Fig. 6d and Supplemental Fig. 5d). While at 4 weeks post-dose 1, responses were low in vaccinated calves without pre-existing Abs, titers in calves with pre-existing Abs were similar between the placebo and vaccine groups. This suggest that the nAb responses in the latter vaccine group largely comprised pre-existing Abs derived from the Ab-positive colostrum. The titers in both vaccinated groups increased after the second dose, but were two- to three-fold lower in the calves which received Ab-positive colostrum (GMTs with versus without pre-existing Abs, at 2 or 4 weeks post-dose 2: 436 versus 1323, and 382 versus 725, respectively). Our data therefore indicate that the pre-existing bRSV Abs may have dampened the vaccine-induced nAb response, consistent with observations made for different vaccines in human infants with maternal Abs^40,41^. However, despite the potential blunting effect of pre-existing responses on the humoral vaccine immunogenicity, the clinical efficacy upon bRSV challenge appeared to be uncompromised, as described above (see Fig. 5).

Upon challenge, titers increased across all groups in both studies. Of note, among the short-DOI groups in Study 1, titers were lower in the single-dose group than in the two-dose group at pre-challenge (GMTs: 58 versus 316, respectively), but similar between these groups two weeks after the challenge (Fig. 6a and Supplemental Fig. 5a). Among the two-dose groups, post-challenge titers were comparable between the short- and long-DOI groups (Fig. 6a, b and Supplemental Fig. 5a, b). A similar trend of comparable post-challenge titers among groups was seen in Study 2. Indeed, the low titer in the single-dose group at pre-challenge increased by 53-fold upon challenge, whereas fold-increases post-challenge were lower in the two-dose groups without or with pre-existing Abs (4.3-fold and 1.5-fold, respectively; Fig. 6c, d and Supplemental Fig. 5c, d).

## Discussion

A pediatric vaccine to protect infants against RSV-linked LTRD is urgently needed to cover the large burden of disease in infants. Vector-based RSV vaccines, such as the ChAd155-RSV candidate vaccine based on a chimpanzee-derived Ad-vector, elicit immune profiles that have not been associated with ERD, and may be a suitable solution for pediatric use. We have evaluated the immunogenicity, safety (in terms of ERD) and vaccine efficacy of the Chad155-RSV pediatric vaccine in a calf model, and studied different settings and regimens to mimic, as closely as possible, the human infant vaccination scenarios. We demonstrate that ChAd155-RSV was immunogenic in naive young calves, inducing low to modest RSV nAb responses following the first dose which were further increased following the second dose. The two-dose vaccination regimen protected the calves from bRSV-induced clinical disease and lung pathology, and significantly reduced viral loads in the nasopharynx and lung after a DOI of either 4 or 16 weeks. The protection conferred by a single vaccination resembled the protection following the two-dose regimen when applying the short DOI, and was near-complete against general illness, fever, and CLA after the long DOI. However, in the latter case, the effects of the single dose on respiratory rates and lung histopathology were less profound than with two doses and a long DOI. Finally, protection after two vaccinations and a short DOI was similar in calves with versus without pre-existing maternal RSV Abs.

Multiple studies have demonstrated that replication-incompetent ChAd vectors against several viruses, including RSV and SARS-CoV-2, are capable of inducing humoral and cell-mediated responses in humans, with acceptable safety profiles^16,19,24,25,42^. Activation of both arms of adaptive immunity is considered critical for protection against severe RSV disease, especially in infants and older adults^11,43–46^. Previously, ChAd155-RSV was demonstrated to induce RSV nAbs and T cell responses in seropositive adult humans^22^. Here we show that two doses of ChAd155-RSV induced robust nAb responses that reached the estimated protective titer in human infants^47^ (i.e., 256) and just exceeded the threshold of 6 log2 that is associated with a reduced risk of hospitalization in infants^48^. The immune responses observed here in calves, which were sustained during the 4-week study period, almost completely protected the animals from bRSV clinical disease and lung pathology, as did the two-dose regimen after the 16-week period. The latter is remarkable, considering that at challenge these nAb responses had decreased to a level (geometric mean of 101) well below the above mentioned protection threshold^47^. Interestingly, this data also suggests that the nAb titer before challenge impacts the magnitude of the Ab response to viral infection, such that higher titers before infection exhibit a lower increase after infection. A likely explanation is that a more robust nAb response will better contain viral replication, and the resulting lower viral levels will then induce a lower increase in Ab titer.

In animals receiving a single vaccine dose, the general illness, fever and lung consolidation readouts, and the virus concentrations in the lung and nasopharynx, were all comparable after long- and short-DOI. However, among these single-dose groups, respiratory rates, and lung histopathological scores with a long DOI (Study 2), while still being lower than the controls, were less reduced than with the short DOI (Study 1). In the absence of a two-dose long-DOI group in the same study, it remains inconclusive whether this slightly compromised protection was due to the more severe challenge condition, or to a lower efficacy of this single-dose regime. Nevertheless, the overall results suggest that a single-dose regime also merits consideration in clinical development (NCT03636906).

Though not measured here, several lines of evidence suggest the presence of vaccine-induced immunity beyond the detected nAb responses. Indeed, in line with the intended pediatric use of ChAd155-RSV, calves received their first injection at a young age. At this age, the bovine immune system is considered not fully mature, resulting in weak nAb responses due to the induction of low-frequency and short-living plasma-cell responses^49–51^. In human infants, differentiation of B cells into long-lived plasma cells after antigenic exposure is known to be reduced due to lack of B cell survival signals in the bone marrow^52–54^. While a similar effect is expected to have occurred in the vaccinated calves, they nevertheless exhibited the typical kinetics of a B cell response to vaccination, in which nAb titers elicited by the first dose were boosted by the second vaccination, and further boosted by the challenge. This suggests that the vaccination had successfully induced plasma-cell and memory B cell responses targeting RSV A Long. Moreover, the fact that all calves in the single-dose groups were strongly protected, with reduced viral titers even though their nAb titers were low, indicated that other arms of immunity, such as T cells, may also have contributed to this level of protection. Indeed, the M2-1 and N proteins are included in the vaccine construct to elicit T cell immunity, and preliminary analysis in a different calf study showed that ChAd155-RSV induced both CD4^+^ and CD8^+^ T cell responses to RSV proteins following a single injection (unpublished data). In addition, T cell responses were detected in the vaccinated adult humans^22^.

A large proportion of the vaccine target population of young infants will have maternal-derived RSV Abs due to natural infection of the mother, and possibly increased levels due to future maternal RSV vaccination^4,55^. Moreover, many infants may receive one of the forthcoming prophylactic anti-RSV monoclonal antibodies (mAbs)^56,57^. Importantly, although our results suggest that the presence of maternal Ab may have had some negative impact on the levels of the nAb response, as observed after the second dose, this did not appear to have affected the protection against the bRSV challenge conferred to these animals. The collective data thus suggest that the presence of pre-existing RSV Abs might have at most minimal effects on the efficacy of ChAd155-RSV in human infants. Nevertheless, future research could help determining which RSV F epitopes are the main targets of the humoral immune response, and whether the observed negative effect of pre-existing Ab has any impact on vaccine immunogenicity in humans.

The more severe clinical outcome in Study 2 compared to Study 1 was unexpected, considering the potential advantageous role of the colostrum (with or without specific immunity), which was only provided to the calves in Study 2. Bacterial examination (by bacterial culture and/or PCR) of BAL and lung tissue samples did not reveal any evidence for an invasion of pathogenic bacteria that might explain this outcome. However, considering the (~3.2 times) higher dose of challenge virus in Study 2 versus Study 1, the more severe clinical data post-challenge might be explained by a challenge dose-response effect. Such effect has also been observed in a (unpublished) in-house challenge-dose titration study. In that study, a (0.7 log_10_) higher challenge dose clearly changed the kinetics of the infection, and resulted in an earlier onset and more rapid development of (more) severe symptoms, without affecting the lung viral loads.

The higher CLA scores of controls in Study 2 may also be related to the more severe challenge condition. However, this difference between studies may have been exacerbated by the fact that the mean CLA score at necropsy was calculated on data from all animals, including those that were terminated 5 or 6 days earlier, when they most likely have been at their disease peak. Indeed, the overall higher CLA scores in the pre-terminated versus surviving animals is consistent with data showing a trend of an association between disease severity scores and CLA scores^30^. Similarly, histopathology scores were also higher in Study 2, though for these scores no difference was found between pre-terminated and recovered animals. This may be because the recovery from such microscopic symptoms generally takes longer. Nonetheless, the two-dose (short-DOI) regimens in groups without pre-existing responses displayed similar efficacies across the two studies. This indicated that the efficacy data from Study 2 can be used to support the overall conclusion on ChAd155-RSV vaccine.

Beyond its utility in RSV vaccine immunogenicity/efficacy studies, the calf model is also suitable for evaluations of vaccine safety including the risk of ERD, a major focus in pediatric RSV vaccine development. In calves, this phenomenon has been characterized by advanced and exacerbated clinical symptoms upon bRSV challenge relative to control bRSV-challenged animals, and by Th2-skewed cytokine profiles, IgE Ab production, pulmonary eosinophilia, and a high ratio of non-functional Abs to nAbs^30,37,38,58–60^. Apart from the relatively small sample size (due in part to the husbandry requirements for cattle), the absence of a positive control vaccine (bovine FI-RSV) inducing ERD may therefore be a potential limitation of our studies. This was done considering that the current research work was not designed as a safety study to extensively investigate all ERD signs or characteristics. Moreover, bovine FI-RSV studies are often hampered by technical hurdles in the preparation of these vaccines, leading to variable vaccine formulations and inconsistent reproduction of enhanced disease (across different studies or across bovine FI-RSV-vaccinated calves within the same study)^30,61,62^. Nonetheless, we observed that all ChAd155-RSV-vaccinated, bRSV-challenged young calves were either free of clinical/pathological symptoms, or exhibited symptoms that were much less severe than in the PBS-injected and bRSV-challenged control groups, while in case of an ERD response the symptoms in vaccinated animals would have been more severe than in the controls. In conclusion, our observations suggest that the risk that ChAd155-RSV will induce ERD in human infants is extremely low, supporting the further clinical development of this vaccine.

## Methods

### Ethical clearance

Prior to the studies’ initiation, protocols were submitted for ethical review and approved by GSK’s ethical committee (approval no: S001698 for Study 1, S003976 for Study 2). Both studies were conducted in Wageningen University and Research (WUR; Wageningen Bioveterinary Research institute, Lelystad, The Netherlands) in accordance with the Dutch Law on Animal Experiments and the European legislations and guidelines (2010/63/EG and ETS 123). Study 1 was authorized by the Animal Ethics Committee of the Animal Sciences Group of WUR. Study 2 was licensed by the Dutch Central Authority for Scientific Procedures on Animals (no. AVD401002015194) and approved by WUR’s Animal Welfare Body.

### Animals and husbandry

Because of the endemic status of bRSV, calves were transported from conventional dairy farms to the institute within 2 h after spontaneous birth. Upon arrival in the facility, animals were housed in individual pens with straw bedding (1.4 m^2^ (Study 1) and 2.2 m^2^ (Study 2) per animal; max. 9 calves/room). The staff applied a strict clothing and hygiene regimen. From ~5 weeks of age, the calves were housed group-wise, first in pens on concrete floor and rubber mats, then, during the challenge phase, in separate pens by group without physical contact between groups. Floor space was adjusted by age (from 3.4 to 4.8 m^2^/calf at 5 and 28 weeks of age, respectively). Rooms were temperature- and humidity-controlled (10–24 °C; 30–80%) with HEPA-filtered ingoing air. Animals were fed at least twice daily. During the first 2–3 weeks of life, they received milk three times daily, and they were weaned at ~9 weeks of age. From 2 to 3 weeks of age, they also received a corn mix followed by hay, grass pellets and concentrates. Drinking water was supplied ad libitum.

The calf populations consisted of 72% males and 18% females in Study 1 (*n* = 39), and of 60% males and 40% females in Study 2 (*n* = 45). They were in majority dairy (Holstein) calves. Randomization in both studies was performed while allowing for an even distribution of gender, age, and breed across the treatment groups.

### Study designs

Two placebo (PBS)-controlled calf studies were performed according to the design displayed in Fig. 1 and detailed in Table 1. Upon arrival in the animal facilities, calves in Study 1 were kept colostrum-deprived, and calves in Study 2 received a single feeding with either bRSV-Ab-negative or bRSV Ab-positive colostrum within 6 h after birth. The colostrum was pre-collected and frozen on Scandinavian (bRSV Ab-negative) or Dutch (bRSV Ab-positive) farms, and screened for (n)Abs and IgG content prior to feeding. The bRSV Ab-positive colostrum used was pooled just prior to the actual recruitment of the newborn calves. All these calves were fed by stomach tube with 3–4 L of Ab-negative colostrum or 3.2 L of Ab-positive colostrum, directly upon arrival. Anti-bRSV antibody levels in calf sera obtained 1 day post colostrum feeding showed a range of 8.3–9.3 log_2_ ELISA antibodies and 4.0–6.0 log_2_ bRSV nAbs. In calves with or without maternal-derived antibodies, the individual serum total protein concentrations varied from 52–67 g/L and 48–79 g/L, respectively (diagnostic reference value: 55–85 g/L).

In Study 1, newborn calves (*n* = 7 or 8 per group; Table 1) tested negative for anti-bRSV Abs were injected either once or twice (4 weeks apart) with ChAd155-RSV or PBS. Challenge occurred either 4 weeks or 16 weeks after the last vaccine dose (referred to as the short or the long DOI), on the same calendar day across groups.

In Study 2, two groups of 9 calves each were fed bRSV Ab-positive colostrum as described above, and received two doses (4 weeks apart) of either ChAd155-RSV or PBS. A third group (*n* = 9) was fed bRSV Ab-negative colostrum and received two doses of ChAd155-RSV, serving to bridge Studies 1 and 2 and as a bRSV Ab-negative control. All three groups were challenged with bRSV after the short DOI, to evaluate the impact of pre-existing RSV Abs on vaccine immunogenicity and efficacy. Two other groups of 9 calves each in Study 2 were fed Ab-negative colostrum, and received one dose of either ChAd155-RSV or PBS. These animals were challenged with bRSV after the long DOI. In Study 2, a total of eight animals in both placebo groups reached humane endpoints (as defined in “Clinical assessments” below) before termination of the study and were euthanized due to severe clinical signs of bRSV infection (see “Results”).

Blood samples were collected at different time points pre- and post-vaccination and post challenge to assess vaccine immunogenicity. Upon bRSV challenge, clinical vaccine efficacy was evaluated through observations of clinical symptoms, measurements of rectal temperature; as well as collection of nasopharyngeal brush samples and broncho-alveolar lavage (BAL) samples, to measure viral loads. At dpc 12 or 13, calves were euthanized. Lungs of all animals were subjected to post-mortem examination to evaluate lung macroscopic and microscopic lesions, focusing on macroscopic CLAs and microscopic pathological changes. For the pre-terminated control animals in Study 2, these examinations were performed at dpc 7 or 8.

### ChAd155-RSV vaccine and bRSV challenge stock preparation

The ChAd155-RSV investigational vaccine consists of a recombinant replication-defective ChAd155 vector construct, engineered to express the RSV F protein deleted of the transmembrane region, and the RSV N and M2-1 proteins, as described previously for this antigen when inserted in a different simian adenoviral vector^63^. The F protein sequence is a consensus sequence, derived from the alignment of different subgroup A isolates retrieved from the National Centre for Biotechnology Information database. The transmembrane and C-terminal domains of the F sequence were deleted, and no pre-fusion stabilizing mutations were included. All antigens were codon-optimized for expression in eukaryotic cells. For the challenge, an in vivo-passaged bRSV (strain Odijk, subtype A, passage 6) was used, which was originally isolated from a calf in the field suffering from acute respiratory disease^64^. The in vivo passage was performed in cesarian-derived specific-pathogen-free calves. BALs used for experimental challenge contained at least 10^2.5^ TCID_50_/mL bRSV, and were tested free from major bovine respiratory viruses, mycoplasmas, and bacteria, by evaluating three in vitro passages on sensitive cell cultures and classical bacterial and mycoplasma culture.

### Vaccination and bRSV challenge

In both studies, calves were vaccinated intramuscularly in the pre-scapular area with 5×10^10^ viral particles of ChAd155-RSV in a 2 mL volume. Four or 16 weeks post-last immunization, calves were challenged by aerosolized inoculation of 2 mL bRSV (strain Odijk, doses: 3.2 log_10_ TCID_50_ in Study 1, and 3.7 log_10_ TCID_50_ in Study 2) using an airbrush (Harder & Steenbeck), using a method described by Antonis et al.^30^. No metaphylaxis was used during the bRSV challenge phase.

### Clinical assessments

Upon challenge, bRSV-related symptoms were monitored daily by the same veterinarian, who was blinded for the treatment groups. Clinical scores for general illness (including depression and loss of appetite), upper respiratory tract disease (URTD; nasal and ocular discharge and cough) and LRTD (increased breathing effort) were assessed according to the scoring system outlined in Supplemental Table 1. The LRTD score was partially based on the actual respiratory rate, which was counted by visual inspection. In addition, rectal temperatures were measured twice daily after the challenge. Humane endpoint criteria were defined as persistent severe depression (max. 24 h), persistent severe dyspnea e.g., respiratory rate of >100 breathings/minute (max. 48 h), or non-reversible respiratory failure such as intermittent open mouth breathing or frothing (immediately).

### Sample collection

Blood samples for serology (8 mL) were collected at different time points (see Fig. 6) by venipuncture of the *V. jugularis*. Just prior to challenge and on dpc 1 to 9, nasopharyngeal samples were collected using sterile nylon bristle brushes (MW126, Medical Wire and Equipment Co. Ltd). Following sampling, brushes were directly agitated in 3 mL transport medium, consisting of EMEM (GIBCO), supplemented with 2% antibiotics and 2% fetal bovine serum (FBS). BAL samples were collected 6 days prior to challenge and on dpc 5, 7, and 9 from all calves (non-sedated) regardless of clinical presentation. BAL fluid was obtained from the caudal part of the lung after instillation of approximately 100 mL D-Phosphate Buffered Saline (GIBCO) via intubation of the ventral nasal meatus. Fluid recovered from the lung (50-80% of the instilled volume) was directly supplemented with 5% FBS. On the days of BAL sampling, clinical disease scores were assessed at least 2 h after sample collection, to prevent the interference of any potential transient effect of the BAL procedure on the clinical presentation.

### Processing of samples for viral load

Nasopharyngeal brush and BAL samples were kept on melting ice immediately following the sampling procedure. In the laboratory, brushes were removed from the medium. Samples were centrifuged for 10 min at 4 °C, at 1300 × *g* or 250 × *g* for nasopharyngeal and BAL samples, respectively. Supernatants were stored at −70 ± 10 °C until further analysis.

### Necropsy and pathological analysis

On dpc 12/13 (or day 7/8 for pre-terminated animals), calves of each treatment group were euthanized by pentobarbital overdose and exsanguination. The lungs were removed, and dorsal and ventral images were taken to calculate the extent of macroscopic lesions (Image Pro Premier 64-bit software). The CLA is presented as percentage of the total lung area. Lung samples were collected, if applicable, on a transition between normal and consolidated tissue, stored in 10% neutral buffered formalin, and embedded in paraffin. For histological examination, 5 μm sections of the right apical and cardiac lobes and the left cardiac lobe were stained with hematoxylin and eosin. Each tissue section was scored from 0 (absent) to 4 (severe) for the following categories of histopathological changes according to the scoring table (Supplemental Table 2): endo-bronchi(oli)tis, peri-bronchitis/vasculitis, interstitial pneumonia and alveolitis. CLA calculations and histological examination (by a European College of Veterinary Pathologists board-certified veterinary pathologist) were performed blinded, as a single independent assessment.

### Viral load assessment

All nasopharyngeal brush and BAL samples were first analyzed by bRSV qPCR (details in Supplementary Information file) to determine the course of viral replication. Subsequently, a selection of these samples collected on various time points was also analyzed by virus titration. Infectious virus titers were determined in duplicate by end-point titration of 10-fold sample dilutions on immortalized embryonic bovine tracheal cells (produced at Wageningen Bioveterinary Research [WBVR], Lelystad, The Netherlands), seeded in 96-well plates. Duplicate plates were incubated for 6–7 days at 37 °C and 5% CO_2_. At the end of the incubation period, virus-infected cells were detected with an anti-F bRSV mAb (“mAb3,” produced at WBVR, diluted 1:100), followed by rabbit anti-mouse antibody conjugated with horseradish peroxidase (Dako P0260, 1:200) followed by chromogenic substrate AEC (Sigma A-6926)^65,66^. For each duplicate plate, the virus titer was determined, and expressed as the endpoint titer at which 50% of the cell monolayers was infected, as calculated according to Reed and Munch approach. The detection limit was 6.3 TCID_50_/mL. Samples tested negative were assigned a value of 2 TCID_50_/mL.

### Neutralization assay

Briefly, decomplemented sera were serially diluted twofold in DMEM medium with 3% FBS, 50 µg/mL gentamicin and 200 mM L-Glutamine; RSV medium). Eight serial dilutions were tested for each serum. Samples and positive control dilutions were mixed with human RSV A Long (ATCC, VR-26) diluted to approximately 100 plaque-forming units/well, and incubated for either 20 min at 33 ^o^C (Study 1) or 120 min at 35 °C (Study 2). After incubation, the virus–serum mixture was transferred to Vero (ATCC, CCL-81) cell-seeded plates, with virus-only wells as 100% infectivity control, while two wells received no virus or serum (cell controls). Plates were incubated for 2 h at 35 °C, then the medium was removed and RSV medium containing 0.5% carboxymethylcellulose (low viscosity) was added to all wells. The plates were incubated for 36 h at 33 °C before staining. Staining was performed with a goat anti-RSV polyclonal antibody (Meridian #B65860G, diluted 1:400) followed by rabbit anti-goat immunoglobulin G (IgG) fluorescein isothiocyanate-conjugated (FITC; Millipore #AP106F, diluted 1:1000; study 1) or rabbit anti-goat IgG-horse radish peroxidase (HRP)-conjugated (Rockland 605-403-B69, diluted 1:1000, study 2). Briefly, cell monolayers were washed with PBS and fixed with 1% paraformaldehyde. After blocking with 2% powdered skim milk/PBS, RSV-positive cells were detected using the goat RSV antiserum followed by the rabbit anti-goat IgG conjugate, diluted in blocking buffer containing a final concentration of 0.01% Evans Blue as counter stain. When the HRP conjugate was used, after Ab staining, True Blue substrate (KPL #5510-0049) was added to all wells to reveal the infectious foci. Plates were scanned using an AxioVision reader and software. Reciprocal nAb titers were expressed in ED60, determined as the inverse of the serum dilution causing 60% reduction in the number of plaques as compared to the control wells (virus only, no serum).

### Statistical analyses

All data were analyzed using analysis of variance models with the appropriate variance and co-variance assumptions and without considering multiplicity of comparisons, in SAS 9.4. The models either considered only group as fixed effect for necropsy endpoints and AUC data or group, time, and the interaction between time and group as fixed effects for endpoints with repeated measures (nAbs and viral loads). Group comparisons were assessed by two-sided *t* tests on estimates computed from those models with alpha level set to 0.05.

Missing values due to premature deaths on study 2, day 124, were treated as such for nAbs and viral loads endpoints.

For clinical endpoints, Areas Under the Curve (AUC) were computed from post challenge data for each animal. To avoid sample size reduction, missing values due to premature deaths in placebo groups from study 2 were replaced by the animal’s average from dpc 5 to its death and AUC value for general illness in healthy animals (AUC = 0) was set to 1.26. For temperature, 37.5 °C (observed minimum) were subtracted from the individual values before AUC calculation.

For necropsy endpoints, data of pre-terminated animals were included in the modeling.

AUC, Nabs and viral loads data were analyzed on the log10 scale and ratios were computed for group comparisons while necropsy endpoints data were untransformed, and differences were computed for group comparisons. Group comparison results are provided as Supplementary Data 1 for Study 1 and Supplementary Data 2 for Study 2, with estimated 95% confidence intervals, exact *P* values and degrees of freedom. Note that the confidence intervals displayed on graphs were generated with GraphPad Prism software on observed values, and hence slightly differ from those estimated with the statistical models.

### Reporting summary

Further information on research design is available in the Nature Research Reporting Summary linked to this article.

## Supplementary information

Supplementary Information

Description of Additional Supplementary Files

Supplementary Data 1

Supplementary Data 2

Reporting Summary

## Data Availability

Source data are provided with this paper. Additional data generated in this study are available in the Supplementary Information.

## Source data

Source Data
