# Supplementary material for: ChAd155-RSV vaccine is immunogenic and efficacious against bovine RSV infection-induced disease in young calves
Source: Nat Commun. 2022 Oct 17;13:6142. doi: 10.1038/s41467-022-33649-3 (PMC9575635; doi:10.1038/s41467-022-33649-3)
Supplement: Supplementary file 1 — Supplementary Information [file 41467_2022_33649_MOESM1_ESM.pdf]

Manuscript NCOMMS-21-34494B

Supplementary Information

ChAd155-RSV vaccine is immunogenic and efficacious against bovine RSV infection-induced disease in young calves

Rineke de Jong[1], Norbert Stockhofe-Zurwieden[1], Judith Bonsing[1], Kai-Fen Wang[2,5], Sarah Vandepaer[3], Badiaa Bouzya[4], Jean-François Toussaint[4,6], Ilse Dieussaert[4], Haifeng Song[2,7*], Ann-Muriel Steff [2*]

Supplemental Table 1 Scoring clinical diseases

Score*	General Illness	Upper Respiratory Tract Disease	Lower Respiratory Tract Disease
0 (Absent)	Bright, alert<br><br>Normal appetite<br><br>Normal behavior	No nasal or ocular discharge.<br><br>No coughing.	Normal respiration<br><br><50 breaths per minute
1 (mild)	Reduced responsive-ness<br><br>Decreased appetite<br><br>Otherwise normal behavior	Nasal or ocular discharge: intermittent watery-mucoid<br><br>Occasional spontaneous dry cough or induced unproductive cough	Abnormal respiration<br><br>51-70 breaths per minute
2 (Moderate)	Depressed, lethargic<br><br>Decreased appetite<br><br>Retreats	Increased nasal or ocular discharge: persistent mucoid-mucopurulent<br><br>Spontaneous productive cough or induced productive cough	Abnormal respiration-obvious abdominal breathing<br><br>71-100 breaths per minute
3 (Severe)	Depressed, soporific<br><br>Anorexia<br><br>Unstable to stand without assistance	Severe nasal or ocular discharge: persistent purulent-hemorrhagic discharge<br><br>Frequent spontaneous productive cough or prolonged productive cough when induced	Abnormal respiration-severe abdominal breathing<br><br>Dyspneic (e.g., mouth breathing/frothing)<br><br>>100 breaths per minute

*Animals under evaluation must show at least one of the described disease characteristics to receive the applicable severity score

Supplemental Table 2 Scoring histopathology

Category	0	1	2	3	4
**bronchitis**	absent	one or few small foci (in at least 1 MF)	small- to medium-size foci (in at least 3 MF)	frequent and/or moderately sized foci (in > 3 MF)	extensive to confluent foci affecting most/all of the tissue (in > 3 MF with bronchial obstruction)
**peribronchitis/ -vasculitis**	absent	one or few small foci (< 10% in at least 1 MF)	small- to medium-size foci (< 10% in at least 3 MF)	frequent and/or moderately sized foci (< 10% in > 3 MF or 10 - 30% affected in at least 1 MF)	extensive to confluent foci affecting most/all of the tissue (>10% in > 3 MF or >30% in at least 1 MF)
**interstitial pneumonia**	absent	one or few small foci (< 10% in at least 1 MF)	small- to medium-size foci (< 10% in at least 3 MF)	frequent and/or moderately sized foci (< 10% in > 3 MF or 10 - 30% affected in at least 1 MF)	extensive to confluent foci affecting most/all of the tissue (>10% in > 3 MF or >30% in at least 1 MF)
**atelectasis**	absent	one or few small foci (< 10% in at least 1 MF)	small- to medium-size foci (< 10% in at least 3 MF)	frequent and/or moderately sized foci (< 10% in > 3 MF or 10 - 30% affected in at least 1 MF)	extensive to confluent foci affecting most/all of the tissue (>10% in > 3 MF or >30% in at least 1 MF)
**alveolitis**	absent	one or few small foci (< 10% in at least 1 MF)	small- to medium-size foci (< 10% in at least 3 MF)	frequent and/or moderately sized foci (< 10% in > 3 MF or 10 - 30% affected in at least 1 MF)	extensive to confluent foci affecting most/all of the tissue (>10% in > 3 MF or >30% in at least 1 MF)

MF: microscopic field at 5x objective magnification.

Supplemental Figure 1. Macroscopic lung consolidation. Representative images of the macroscopic lung consolidation found in Study 1 (**a, b**) and Study 2 (**c–e**) are shown. (**a**–**d**) Lungs were collected from calves without pre-existing antibodies that were challenged after a 4-month duration of immunity, and were terminated at study completion on day post challenge (dpc) 12 or 13. (**e**) Lungs were collected from a calf with pre-existing antibodies that was challenged after a 1-month duration of immunity, and was pre-terminated on dpc 8 while being compliant to pre-defined humane endpoint criteria. The calves had received either two doses of placebo (**a**), two doses of ChAd155-RSV (**b**), one dose of placebo (**c**), one dose of ChAd155-RSV (**d**), or two doses of placebo (**e**).

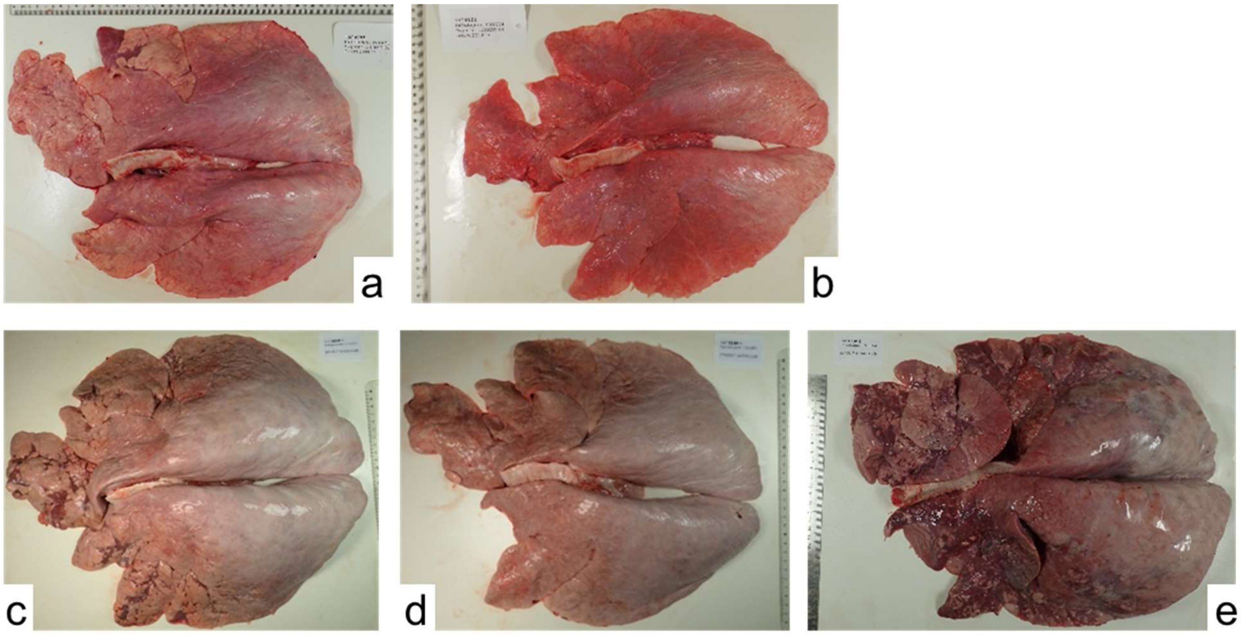

Supplemental Figure 2. Lung histopathological changes. Representative images of lung histology are shown. (**a**) Normal histology with unchanged bronchi and alveoli at day post challenge (dpc) 12, in a calf without pre-existing antibodies that had received two doses of ChAd155-RSV and was challenged after a 4-month duration of immunity (DOI), in Study 1. (**b**) Moderate pneumonia with partially obliterating bronchitis and focal moderate alveolitis with infiltration of neutrophils and mononuclear inflammatory cells. The tissue was collected at dpc 13, from a calf without pre-existing antibodies that had received one dose of ChAd155-RSV and was challenged after a 4-month DOI, in Study 2. (**c**) Severe broncho-pneumonia with obstruction of bronchi by epithelial debris, mucus and inflammatory cells, and extended infiltration of granulocytes and lymphomonocytic inflammatory cells in alveoli. The tissue was collected at dpc 8, from a calf with pre-existing antibodies that had received two doses of placebo, in Study 2. The histological examination was performed blinded by a ECVP board-certified veterinary pathologist as a single independent assessment. All slides were hematoxylin–eosin-stained, and are shown at 10× objective magnification, with horizontal bars indicating 100 µm. Bronchi and alveoli are indicated by arrows and stars, respectively.

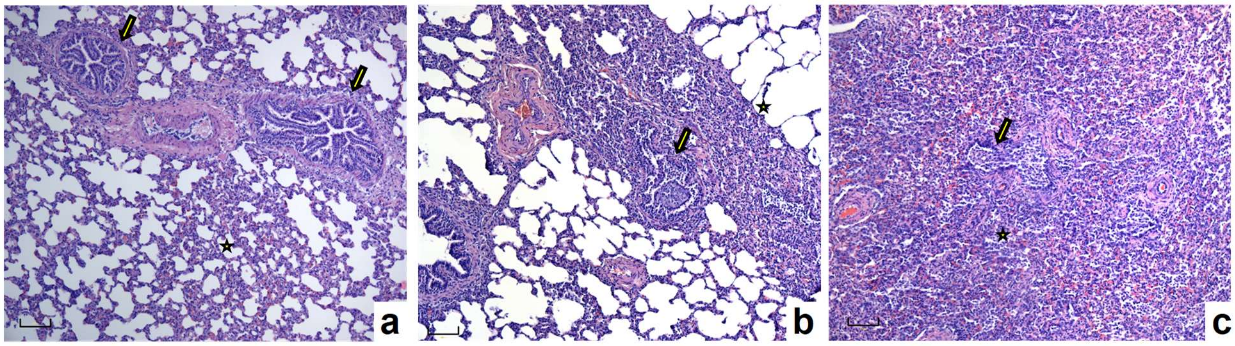

Supplemental Figure 3. Lung histopathology composite scores by category. Study groups received either a single dose (1D) (**a, c**) or two doses (2D) (**a, b, d**)of ChAd155-RSV vaccine or placebo (saline). Each treatment was followed by either a short (4 weeks) (**a, d**) or long (16 weeks) (**b, d**) duration of immunity (short DOI or long DOI, respectively), and then a bovine RSV (bRSV) challenge. Study groups in (**d**) included calves without (Ab-) or with (Ab+) maternal anti-bRSV antibodies. Pathology scores for alveolitis, interstitial pneumonia, peribronchitis and bronchitis were determined based on microscopic analysis of the lung pathological changes upon the bRSV challenge. Composite scores were derived by summing the scores per category across all animals in the study group.

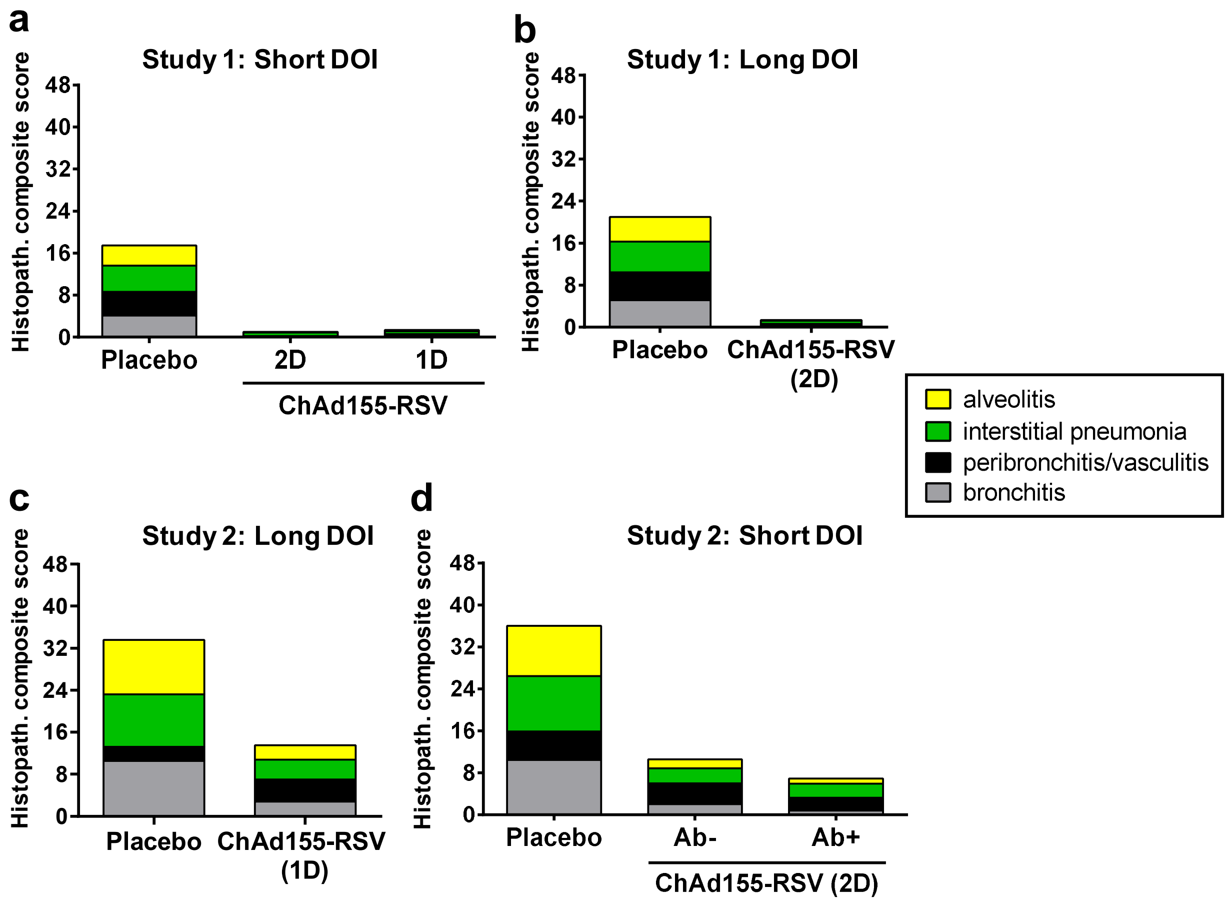

Supplemental Figure 4: RSV load determined by qPCR. qPCR was performed on BAL samples (dpc -6, 5, 7, 9) (**a, c, e, g**) and nasopharyngeal swabs (day post-challenge [dpc] 0-9) (**b, d, f, h**). Total nucleic acids were extracted from 200 µL sample using the MagNA Pure LC Total Nucleic Acid Isolation Kit (Roche). The conserved bRSV N-gene was detected with a primer/taqman probe mix (forward primer CAGGATGTGAATGGA, reverse primer CCTTGAACTTCTGATG and probe ATGTTAGCACTTCA) by real-time RT-PCR using the QuantiFast Multiplex Kit (Qiagen) according to the manufacturer's instructions. All assays included reverse transcription of RNA into cDNA and were run on the Applied Biosystems 7500 under optimized cycling conditions (40 cycles). Threshold cycle (Ct) values were calculated and are displayed graphically over time as mean values with 95% confidence intervals. Of note, dpc4 nasal samples were not available due to a technical failure and sample sizes of the placebo groups in Study 2 had decreased on both dpc 8 (16 week [w] group: n=6; 4w group: n=8) and on dpc 9 (16w group: n=4; 4w group: n=6).

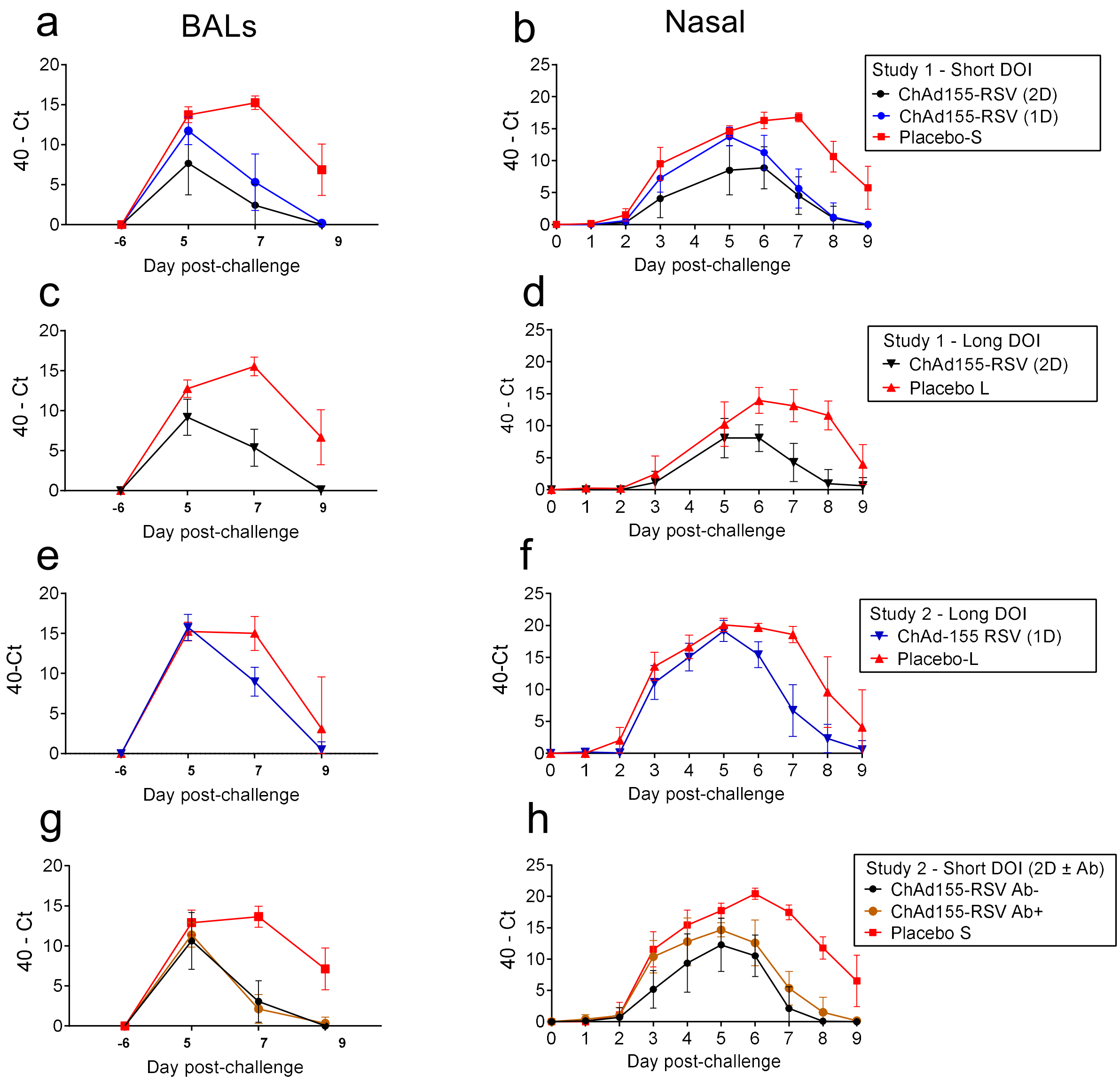

a
BALs
b
Nasal
Study 1 - Short DOI
ChAd155-RSV (2D)
ChAd155-RSV (1D)
Placebo-S
c
d
Study 1 - Long DOI
ChAd155-RSV (2D)
Placebo L
e
f
Study 2 - Long DOI
ChAd-155 RSV (1D)
Placebo-L
g
h
Study 2 - Short DOI (2D ± Ab)
ChAd155-RSV Ab-
ChAd155-RSV Ab+
Placebo S
40 - Ct
40-Ct
Day post-challenge

Supplemental Figure 5. Individual human RSV A-specific nAb titers. Human RSV subtype A (hRSV A) neutralizing antibody (nAb) titers are presented. Titers are expressed as the inverse of the serum dilution causing 60% reduction in the number of plaques as compared to the virus control wells (ED_{60}). Geometric means (solid lines) and individual nAb titers for each group are color coded according to the keys in each graph. Calves (n= 7 to 9 biologically independent animals, see Table 1) received a single or two doses (1D or 2D, respectively) of ChAd155-RSV vaccine or placebo, followed by a short (4 weeks, panels **a** and **d**) or long (16 weeks, panels **b** and **c**) duration of immunity (short DOI or long DOI, respectively), and then a bovine RSV challenge. Placebo-S and placebo-L groups indicate the control groups subjected to the short-DOI and long-DOI regimens, respectively (note that the sample sizes of the placebo-L and placebo-S groups in Study 2 decreased post-challenge; see Figs. 3 and 5, respectively). Groups in (**d**) included calves with or without pre-existing bovine RSV Ab groups (Ab+ or Ab-, respectively). Weeks of study: weeks post-dose 1 for all groups except short DOI-1D, which was injected at week 4 (Study 1). Dotted lines represent the limit of detection (LOD) i.e., 32 ED_{60}. Negative samples were assigned the value of the LOD. Titers of vaccine and control groups were compared using ANOVA mixed models for repeated measurements. Significant differences between vaccine groups and the respective control groups are presented as asterisks color-matched with the vaccine group indicated in the keys (*P <=0.05; ***P <= 0.001). Triangles and crosses below the x-axis denote the time-points of placebo or vaccine injections and the bRSV challenge, respectively. Source data and statistical analyses with exact P values are provided as a Source Data file and Supplementary Data files 1 & 2, respectively.

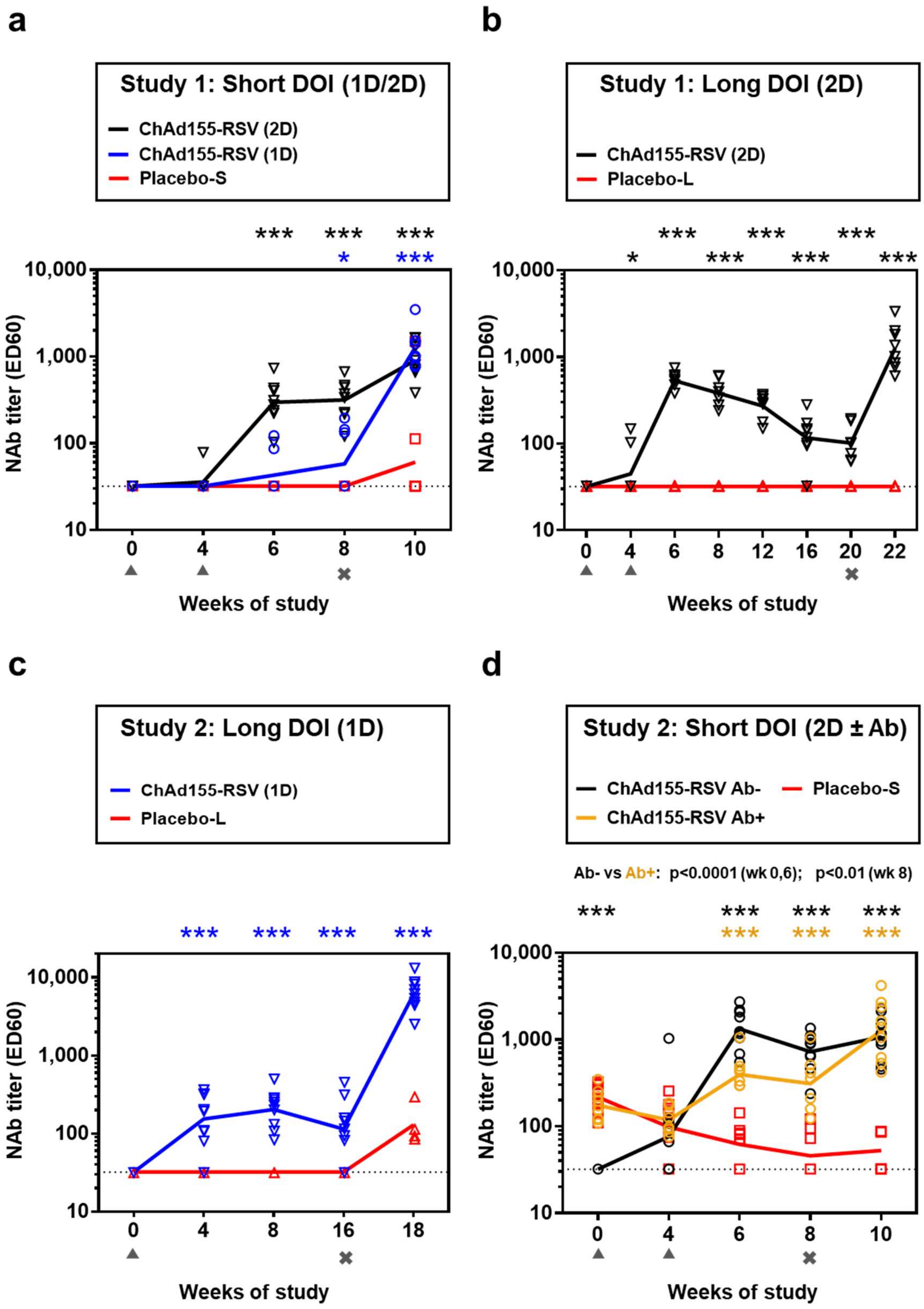
a
Study 1: Short DOI (1D/2D)
ChAd155-RSV (2D)
ChAd155-RSV (1D)
Placebo-S
*** *** ***
* ***
NAb titer (ED60)
10,000
1,000
100
10
0 4 6 8 10
Weeks of study
b
Study 1: Long DOI (2D)
ChAd155-RSV (2D)
Placebo-L
* *** *** *** *** *** ***
NAb titer (ED60)
10,000
1,000
100
10
0 4 6 8 12 16 20 22
Weeks of study
c
Study 2: Long DOI (1D)
ChAd155-RSV (1D)
Placebo-L
*** *** *** ***
NAb titer (ED60)
10,000
1,000
100
10
0 4 8 16 18
Weeks of study
d
Study 2: Short DOI (2D ± Ab)
ChAd155-RSV Ab-
Placebo-S
ChAd155-RSV Ab+
Ab- vs Ab+: p<0.0001 (wk 0,6); p<0.01 (wk 8)
*** *** *** ***
*** *** ***
NAb titer (ED60)
10,000
1,000
100
10
0 4 6 8 10
Weeks of study
