# Supplementary material for: ChAd155-RSV vaccine is immunogenic and efficacious against bovine RSV infection-induced disease in young calves
Source: Nat Commun. 2022 Oct 17;13:6142. doi: 10.1038/s41467-022-33649-3 (PMC9575635; doi:10.1038/s41467-022-33649-3)
Supplement: Supplementary file 2 — Description of Additional Supplementary Files [file 41467_2022_33649_MOESM2_ESM.pdf]

Description of Additional Supplementary Files

File Name: Supplementary Data 1
Description: Detailed statistical results for Study 1

File Name: Supplementary Data 2
Description: Detailed statistical results for Study 2
