# Supplementary material for: ChAd155-RSV vaccine is immunogenic and efficacious against bovine RSV infection-induced disease in young calves
Source: Nat Commun. 2022 Oct 17;13:6142. doi: 10.1038/s41467-022-33649-3 (PMC9575635; doi:10.1038/s41467-022-33649-3)
Supplement: Supplementary file 5 — Reporting Summary [file 41467_2022_33649_MOESM5_ESM.pdf]

nature portfolio

Corresponding author(s): Ann-Muriel Steff
Haifeng Song

Last updated by author(s): Sep 13, 2022

Reporting Summary

Nature Portfolio wishes to improve the reproducibility of the work that we publish. This form provides structure for consistency and transparency in reporting. For further information on Nature Portfolio policies, see our Editorial Policies and the Editorial Policy Checklist.

Statistics

For all statistical analyses, confirm that the following items are present in the figure legend, table legend, main text, or Methods section.

n/a	Confirmed	
☐	☒	The exact sample size (n) for each experimental group/condition, given as a discrete number and unit of measurement
☒	☐	A statement on whether measurements were taken from distinct samples or whether the same sample was measured repeatedly
☐	☒	The statistical test(s) used AND whether they are one- or two-sided<br>*Only common tests should be described solely by name; describe more complex techniques in the Methods section.*
☐	☒	A description of all covariates tested
☐	☒	A description of any assumptions or corrections, such as tests of normality and adjustment for multiple comparisons
☐	☒	A full description of the statistical parameters including central tendency (e.g. means) or other basic estimates (e.g. regression coefficient) AND variation (e.g. standard deviation) or associated estimates of uncertainty (e.g. confidence intervals)
☐	☒	For null hypothesis testing, the test statistic (e.g. F, t, r) with confidence intervals, effect sizes, degrees of freedom and P value noted<br>*Give P values as exact values whenever suitable.*
☒	☐	For Bayesian analysis, information on the choice of priors and Markov chain Monte Carlo settings
☒	☐	For hierarchical and complex designs, identification of the appropriate level for tests and full reporting of outcomes
☒	☐	Estimates of effect sizes (e.g. Cohen's d, Pearson's r), indicating how they were calculated

Our web collection on statistics for biologists contains articles on many of the points above.

Software and code

Policy information about availability of computer code

Data collection	ScanLab/Axiovision for foci counting in bRSV neutralization assay<br>Applied Biosystems 7500 Fast Realtime PCR instrument
Data analysis	SAS version 9.4 for statistical analyses<br>GraphPad Prism software, version 9.3.1 for figure generation and display of central tendency and confidence intervals<br>Applied Biosystems 7500 Fast Realtime PCR instrument, system software for qPCR data analysis<br>Image Pro Premier 64-bit software for evaluation of consolidated lung area

For manuscripts utilizing custom algorithms or software that are central to the research but not yet described in published literature, software must be made available to editors and reviewers. We strongly encourage code deposition in a community repository (e.g. GitHub). See the Nature Portfolio guidelines for submitting code & software for further information.

Data

Policy information about availability of data

All manuscripts must include a data availability statement. This statement should provide the following information, where applicable:

- Accession codes, unique identifiers, or web links for publicly available datasets
- A description of any restrictions on data availability
- For clinical datasets or third party data, please ensure that the statement adheres to our policy

The raw data underlying the figures are available as source data files. The statisitical analyses files with exact P values, degrees of freedom are also provided.

Human research participants

Policy information about studies involving human research participants and Sex and Gender in Research.

Reporting on sex and gender	Not applicable
Population characteristics	Not applicable
Recruitment	Not applicable
Ethics oversight	Not applicable

Note that full information on the approval of the study protocol must also be provided in the manuscript.

Field-specific reporting

Please select the one below that is the best fit for your research. If you are not sure, read the appropriate sections before making your selection.

☒ Life sciences ☐ Behavioural & social sciences ☐ Ecological, evolutionary & environmental sciences

For a reference copy of the document with all sections, see nature.com/documents/nr-reporting-summary-flat.pdf

Life sciences study design

All studies must disclose on these points even when the disclosure is negative.

Sample size	Sample size is described in table 1 of the manuscript and ranged from 7 to 9 animals per group. Although no sample size calculation was statistically performed, this sample size was considered sufficient and in accordance with standards in the field, when performing studies with such large animals. Of note, there is also a limitation in the number of such large animals (> 70 kg) that can be housed at once in a single study.
Data exclusions	No data were excluded from the analysis. When animals were terminated or died before the end of the study, due to severity of symptoms, this is mentioned in the figure legends and the data imputation for deceased animals is explained and provided.
Replication	Firstly, all groups contained at least 7 animals, and variability within groups is demonstrated by error bars and individual values, provided either in the figures or in the source data file. Secondly, the manuscript describes two separate experiments and at least one of the treatment groups was included in both experiments: ChAd155-RSV, 2 doses, challenge 4 weeks post last dose, in the absence of maternal antibodies. Placebo groups were used in both studies. We consider that the results obtained in the treatment group common to the 2 experiments were reproducible. As clearly discussed in the manuscript, the virulence of the challenge was higher in the second experiment but this did not prevent the conclusion on the efficacy of the vaccine.
Randomization	Randomization in both studies was performed, allowing for an even distribution of gender, age, and breed across the treatment groups.
Blinding	Investigator performing clinical examination, histopathology and sample analysis were blinded to the treatment groups. This is indicated in the figure legends when appropriate.

Reporting for specific materials, systems and methods

We require information from authors about some types of materials, experimental systems and methods used in many studies. Here, indicate whether each material, system or method listed is relevant to your study. If you are not sure if a list item applies to your research, read the appropriate section before selecting a response.

Materials & experimental systems

n/a	Involved in the study
☐	☒ Antibodies
☐	☒ Eukaryotic cell lines
☒	☐ Palaeontology and archaeology
☐	☒ Animals and other organisms
☒	☐ Clinical data
☒	☐ Dual use research of concern

Methods

n/a	Involved in the study
☒	☐ ChIP-seq
☒	☐ Flow cytometry
☒	☐ MRI-based neuroimaging

Antibodies

Antibodies used

Antibody used for RSV detection in the neutralization assay:
GOAT ANTI RSV (ALL ANTIGENS) (B65860G) - Meridian Life Sciences; diluted 1:400

Antibody used for bRSV detection in viral load assessments:
"mAb3", diluted 1:100

Secondary antibodies:
Rabbit anti-goat IgG-horse radish peroxidase (HRP)-conjugated - Rockland 605-403-B69, diluted 1:1000
Rabbit anti-goat fluorescein isothiocyanate (FITC)-conjugated - Millipore #AP106F, diluted 1:1000
Rabbit anti-mouse antibody conjugated with horseradish peroxidase - Dako P0260, diluted 1:200

Validation

The antibodies used in this study are widely used and from commercial source:

RSV antibody for neutralization assay: https://collateral.meridianlifescience.com/view/455424416/25/

Secondary antibodies:
https://www.2bscientific.com/Products/Rockland-Inc/605-403-B69/Anti-Goat-IgG-HL-HRP-Conjugated-Pre-Adsorbed
https://www.emdmillipore.com/CA/en/product/Rabbit-Anti-Goat-IgG-Antibody-FITC-conjugate,MM_NF-AP106F
https://www.agilent.com/en/product/specific-proteins/elisa-kits-accessories/rabbit-anti-mouse-immunoglobulins-hrp-solid-phase-absorbed-2717115

One antibody "mAb3' was "home-made" and is described in the following publications:
https://pubmed.ncbi.nlm.nih.gov/9541615/
https://pubmed.ncbi.nlm.nih.gov/9008336/

Eukaryotic cell lines

Policy information about cell lines and Sex and Gender in Research

Cell line source(s)	Vero cells for RSV neutralization assay (ATCC, CCL-81)
Authentication	Not authenticated
Mycoplasma contamination	Cells not tested for mycoplasma contamination
Commonly misidentified lines (See ICLAC register)	None

Animals and other research organisms

Policy information about studies involving animals; ARRIVE guidelines recommended for reporting animal research, and Sex and Gender in Research

Laboratory animals

No laboratory animals were used in the study

Wild animals

Calves were transported from conventional dairy farms to the institute within 2 hours after spontaneous birth. Upon arrival in the facility, animals were housed in individual pens with straw bedding (1.44 m2/animal; 9 calves/room). The staff applied a strict clothing and hygiene regimen. From ~5 weeks of age, the calves were housed group-wise, first in pens on concrete floor and rubber mats, then, during the challenge phase, in separate pens by group without physical contact between groups. Floor space was adjusted by age (from 3.5 to 4.9 m2/calf at five and 28 weeks of age, respectively). Rooms were temperature- and humidity-controlled (10-24°C; 30–80%) with HEPA-filtered ingoing air. Animals were fed at least twice daily. During the first 2-3 weeks of life, they received milk three times daily, and they were weaned at ~9 weeks of age. From 2-3 weeks of age, they also received a corn mix followed by hay, grass pellets and concentrates. Drinking water was supplied ad libitum.
The calf populations consisted of 72% males and 18% females in Study 1 (n=39), and of 60% males and 40% females in Study 2 (n=45). They were in majority dairy (Holstein) calves. Randomization in both studies was performed while allowing for an even

	distribution of gender, age, and breed across the treatment groups. Animals were euthanized at the end of the study to perform a post-mortem examination and evaluate lung pathology. They were euthanized by intravenous injection of an overdose of Pentobarbital followed by exsanguination.
Reporting on sex	Sex used as factor during randomization, no analysis performed by sex.
Field-collected samples	No field collected samples were used in the study
Ethics oversight	Studies were ethically reviewed and approved by GSK's ethical committee (approval no: S001698 for Study 1, S003976 for Study 2). Both studies were conducted in Wageningen University and Research (WUR; Wageningen Bioveterinary Research institute, Lelystad, The Netherlands) in accordance with the Dutch Law on Animal Experiments and the European legislations and guidelines (2010/63/EG and ETS 123). Study 1 was authorized by the Animal Ethics Committee of the Animal Sciences Group of WUR. Study 2 was licensed by the Dutch Central Authority for Scientific Procedures on Animals (no. AVD401002015194) and approved by WUR's Animal Welfare Body.

Note that full information on the approval of the study protocol must also be provided in the manuscript.
